# Nucleotide Diversity Analysis of Three Major Bacterial Blight Resistance Genes in Rice

**DOI:** 10.1371/journal.pone.0120186

**Published:** 2015-03-25

**Authors:** Waikhom Bimolata, Anirudh Kumar, Sai Kiran Reddy M, Raman Meenakshi Sundaram, Gouri Sankar Laha, Insaf Ahmed Qureshi, Irfan Ahmad Ghazi

**Affiliations:** 1 Department of Plant Sciences, School of Life Sciences, University of Hyderabad, Prof. C. R. Rao Road, Gachibowli, Hyderabad, 500046, India; 2 Crop Improvement Section, Directorate of Rice Research, Rajendranagar, Hyderabad, 500030, India; 3 Crop Protection Section, Directorate of Rice Research, Rajendranagar, Hyderabad, 500030, India; 4 Department of Biotechnology and Bioinformatics, School of Life Sciences, University of Hyderabad, Prof. C. R. Rao Road, Gachibowli, Hyderabad, 500046, India; International Rice Research Institute, PHILIPPINES

## Abstract

Nucleotide sequence polymorphisms among *R* gene alleles influence the process of co-evolutionary interaction between host and pathogen by shaping the response of host plants towards invading pathogens. Here, we present the DNA sequence polymorphisms and diversities present among natural alleles of three rice bacterial blight resistance genes, *Xa21*, *Xa26* and *xa5*. The diversity was examined across different wild relatives and cultivars of *Oryza* species. Functional significance of selected alleles was evaluated through semi-quantitative reverse transcription polymerase chain reaction and real time PCR. The greatest nucleotide diversity and singleton variable sites (SVS) were present in *Xa26* (π = 0.01958; SVS = 182) followed by *xa5* and *Xa21* alleles. The highest frequency of single nucleotide polymorphisms were observed in *Xa21* alleles and least in *xa5*. Transition bias was observed in all the genes and ‘G’ to ‘A’ transitions were more favored than other form of transitions. Neutrality tests failed to show the presence of selection at these loci, though negative Tajima’s D values indicate the presence of a rare form of polymorphisms. At the interspecies level, *O*. *nivara* exhibited more diversity than *O*. *sativa*. We have also identified two nearly identical resistant alleles of *xa5* and two sequentially identical alleles of *Xa21*. The alleles of *xa5* showed basal levels of expression while *Xa21* alleles were functionally not expressed.

## Introduction

Plants are constantly exposed to various biotic stresses like insect pests and pathogens and have evolved several mechanisms to guard themselves these stresses. As pathogens challenge the fitness of the plant, they are regarded as natural selective agents shaping evolutionary dynamics of host-pathogen co-evolution [1, 2]. In crops like rice, infections by pathogens influence yield, morphology, grain quality and texture [3]. Bacterial blight (BB) is a major disease of rice caused by *Xanthomonas oryzae* pv. *oryzae*. The annual yield loss by this disease is recorded up to 20–50% [4–6]. At the phenotypic level, symptomatic variations in natural rice populations are generally observed after pathogen infection. This variation may be correlated to the genetic diversity in host plants. Bacterial blight is prevalent and widespread in most of the rice cultivating countries of South and South East Asia including India. For combating the disease, the most effective and economical measure is exploitation of host plant resistance. To date, more than 38 *R* genes for BB resistance have been reported [7, 8]. Diversity analysis of these genes in natural population will facilitate identification of allelic variations which can be exploited in resistance breeding programs [9]. Natural diversity is an important asset for functional genomics studies and crop improvement. Abundant genetic resources available in nature give us an opportunity to explore and discover new forms of resistance genes.

Study of the evolutionary dynamics of disease resistance genes began twenty years ago, using Arabidopsis and tomato as model plants. Recently, various reports on molecular evolution and genetic diversity of blast resistance genes for blast disease caused by *Magnaporthe grisae* are available; however similar studies for bacterial blight resistance genes are limited [10–13]. Among different factors, pathogens play an important role as a selective agent in the evolution of *R* genes. Host and pathogen co-evolve, resulting in continuance of allelic disparity of *R* genes through balanced selection [14]. Sequence polymorphism in naturally occurring alleles may alter the gene expression profile and functional characteristics of proteins leading to phenotypic variations of a trait [15, 16]. Recent studies conclude that nucleotide changes in the non-coding and regulatory sites of *R* genes also contribute to resistance or susceptibility phenotypes of a disease in addition to nucleotide variations in the coding region [17, 18].

In this study, we present the analysis of natural polymorphism with respect to three major bacterial blight resistance genes: *Xa21*, *Xa26* and *xa5*, at the sequence level in a set of rice germplasm. *Xa21* is a broad spectrum bacterial blight resistance gene, originally derived from a wild rice *O*. *longistaminata* and introgressed into cultivated rice [19]. Song et al. [20] reported the cloning of this gene which encodes a LRR threonine rich receptor like kinase domain containing protein. *Xa26* is a dominant *R* gene, which provides resistance against Chinese, Japanese and Korean *Xoo* strains. Both *Xa21* and *Xa26* genes had been mapped on the long arm of Chromosome 11 of rice. The presence of *Xa26* in rice was first reported in Chinese *O*. *sativa* cultivar Minghui 63. Plants with this gene exhibited broad resistance both at seedling and adult stages. *Xa26* also encodes a LRR receptor kinase-type protein [21]. Both *Xa26* and *Xa3* have been shown to be the same gene [22] and is an important gene in the breeding of japonica cultivars with BB resistance in China [23]. *xa5* is a recessive gene, which confers race specific blight resistance against Philippines *Xoo* race 1 (PXO86). The *xa5* gene is a mutant form of rice transcription factor OsTFIIAγ5 (*Xa5*) where there is a substitution variant of a single amino acid: V39E [24]. *xa5* codes for 106 amino acids and both the dominant and recessive genes are constitutively expressed in different tissues of the plant [25]. The predicted 3-D structure of xa5 protein after its superimposition with *Xa5* and TFIIA shows that due to the substitution of V39E, a minor variation occurs in the third helix domain of xa5 protein [25].

Previously, we reported the nucleotide diversity analysis of *Xa27* [13]. The present study was designed to analyze the molecular evolution and nucleotide sequence diversity existing among naturally occurring alleles of rice bacterial blight resistance genes *Xa21*, *Xa26* and *xa5*. The objectives of this study were to (i) determine the DNA polymorphism level of *Xa21*, *Xa26* and *xa5* in different rice germplasms; (ii) to analyze the evolutionary relationship among the alleles of each gene; (iii) to study the type of selection acting at these loci.

## Materials and Methods

### Plant material, screening and DNA isolation

In our previous study, we reported screening of wild and cultivated rice accessions for resistance against set of five Indian *Xoo* isolates DX011, DX133, DX020, DX015 and DX127 [13]. Based on this, we isolated alleles of *Xa26*, *Xa21* and *xa5* genes from rice accessions mentioned in Table 1. Plants were inoculated following Kauffman et al. [26]. Rice plants at booting stage were inoculated with suspension of *Xoo* culture at the concentration of 0.1–0.2 OD (1x10^8^–1x 10^9^ CFU/ml) by leaf clipping method. Three plants (five leaves per plant) were used to inoculate for each isolate. Sterile water was used in place of bacterial suspension for the control plants. Disease spectrum was evaluated after 15 days of inoculation by measuring the lesion lengths and calculating the relative lesion length (RLL), that is the ratios of lesion length to leaf length (RLL). Disease scoring was performed by following Standard Evaluation System for Rice (SES scale) [27]. Genomic DNA was isolated from fresh tender leaves of rice plants following CTAB method [28]. The required chemicals were procured from Sigma Aldrich (USA).

**Table 1:** List of rice accessions used for isolation and nucleotide diversity analysis of alleles.

Species	Code/accession	Origin	Phenotype
*O*. *sativa*	MohemPhou	Manipur, India	R
*O*. *sativa*	MustiLaiphou	Manipur, India	R
*O*. *sativa*	TN1	DRR, Hyd (India)	S
*O*. *sativa*	PB1	DRR, Hyd (India)	S
*O*. *sativa*	R47	DRR, Hyd (India)	MR
*O*. *sativa*	IR20	DRR, Hyd (India)	S
*O*. *sativa*	PAU933	DRR, Hyd (India)	MR
*O*. *sativa*	R4	DRR, Hyd (India)	R
*O*. *sativa*	Kalamekri	DRR, Hyd (India)	R
*O*. *sativa*	AC32753	DRR, Hyd (India)	R
*O*. *nivara*	ON 38–1	DRR, Hyd (India)	R
*O*. *nivara*	M 209 A	DRR, Hyd (India)	R
*O*. *nivara*	81832	DRR, Hyd (India)	R
*O*. *nivara*	106133	DRR, Hyd (India)	R
*O*. *nivara*	210223	DRR, Hyd (India)	R
*O*. *nivara*	81852	DRR, Hyd (India)	R
*O*. *nivara*	100963	DRR, Hyd (India)	R
*O*. *nivara*	102166	DRR, Hyd (India)	R
*O*. *nivara*	ON132	DRR, Hyd (India)	R
*O*. *nivara*	ON15	DRR, Hyd (India)	R
*O*. *longistaminata*	OL1	CRRI, Cuttack (India)	R
*O*. *longistaminata*	OLD	DRR, Hyd (India)	R
*O*. *alta*	IC384116	NBPGR (India)	R
*O*. *officinalis*	IC203740	NBPGR (India)	R

R resistance, S susceptible, MR moderately resistance

### Isolation and sequencing of alleles

Gene specific primers were designed for *Xa21* (U37133.1) [20], *Xa26* (DQ426646.1, cultivar Zhachanglong) [22], and *xa5* (Os05g0107700). The entire coding and non-coding region of each gene was covered using overlapping primers (S1 Table). For *Xa21*, 18 alleles were isolated from *O*. *nivara* (M209A, 81832, 81852, 100963, 102166, 106133, ON132, ON15 and ON34), *O*. *sativa* (IR20, TN1, PB1, PAU933, MohemPhou, MustiLaiphou and R4), *O*. *longistaminata* (OL1 and OLD); 14 alleles of *Xa26*, were amplified from *O*. *nivara* (ON 38–1, M 209 A, 81832, 81852, 106133, 210223 and ON132), *O*. *sativa* (TN1, PB1, MohemPhou, MustiLaiphou and R47), *O*. *alta* (IC384116), *O*. *officinalis* (IC203740); and 15 alleles of *xa5* were amplified from (M209A, 81832, 81852, 100963, 106133, ON132 and ON15), *O*. *sativa* (TN1, PB1, MohemPhou, MustiLaiphou, Kalamekri and AC32753), *O*. *longistaminata* (OL1 and OLD). PCR reactions were carried out using a standard protocol under the following conditions: The total reaction volume was 30μL and the reaction mixture consisted of 50 ng genomic DNA, 0.9 μM of each primer, 0.2 mM of each dNTP, 5 x PCR buffer (10 mM Tris-HCl, pH 8.0, 1.5 mM MgCl_2_), and 0.6 unit (2U/ μL) of High Fidelity Phusion polymerase (Finnzymes, Thermo Scientific USA). Hi-Fi polymerase was used as it has proof reading ability. The PCR program was set for the initial denaturation at 98°C for 3 min, 35 cycles of amplification with DNA denaturation at 98°C for 15 s, and annealing was set at different temperatures for different primers for 30 s. Elongation was kept at 72°C with a different time period specific for the product size. There was a slight variation in the PCR amplification pattern from one primer to another based on the annealing temperature. The amplified product was then separated and resolved on 1% agarose gel and purified using a gel extraction kit (Sigma Aldrich, USA). The purified PCR products were then cloned into pTZ57R/T vector (Thermo Scientific, USA) and sequenced commercially by SciGenome Labs (Cochin, India). Two clones of each allele were sequenced to confirm the accuracy of the sequence. The sequences obtained in this study were submitted to the NCBI GenBank database. The GenBank accession numbers of the alleles are KF689653- KF689667 and KJ890413-KJ890443.

### Allelic diversity and sequence data analysis

The raw sequence data obtained were edited and aligned with the help of the “align two sequences” option of NCBI BLAST (http://blast.ncbi.nlm.nih.gov) and the overlapping nucleotide sequences were removed. The sequenced products of different primer combinations were then arranged into a complete sequence. The coding regions and the UTR regions were determined from the reference sequences (sequences used for designing primers). The alleles obtained for *Xa21*, *Xa26* and *xa5*, respectively, were compared with that of the gene sequence from IRBB21 (possessing *Xa21*), IRBB3 (possessing *Xa3/Xa26*), IRBB5 (possessing *xa5*) and IR24 (possessing susceptible alleles at the three loci, i.e. *xa21*, *xa3/xa26* and *Xa5*). Allelic and nucleotide diversity were then analyzed as follows: the percentage sequence identity between allele and reference sequences were determined by pairwise alignment using NCBI BLAST [29] (http://blast.ncbi.nlm.nih.gov). The sequence variation among alleles was compared using summary statistics ‘π’ (average pairwise difference or average number of nucleotide diversity per site) [30] and ‘θ’ (population mutation parameter or number of segregating sites) [31]. Both inter and intra-specific polymorphisms among alleles of the three genes were analyzed using DnaSP program version 5.1 (http://www.ub.edu/dnasp) [32]. Sequences were first aligned using Clustal Omega (www.clustal.org/omega/‎) [33]. The output aligned file was saved in Fasta format and used as an input file for analysis in DnaSP. Total number of polymorphic sites with single nucleotide polymorphism (SNPs), insertions and deletions (InDel), and synonymous and non-synonymous substitutions were estimated [34]. The analysis was performed with both the coding and non-coding regions. All the diversity analysis was performed as previously described [13]. Briefly, Tajima’s D [35] and Fu & Li’s D [36] were analyzed to determine nature of selection/departure from neutrality. Linkage disequilibrium was estimated based on the parameter of R^2^ between all SNPs using DnaSP v5.10. The significance of LD was measured statistically using Fisher’s exact test and Chi-square test. Decay in LD was determined by plotting graph of R^2^ versus pairwise distance (bp).

### Constructing phylogenetic trees

The phylogenetic trees depicting the genetic relatedness among alleles were constructed for each gene using MEGA 4 [37] (www.megasoftware.net/mega4/mega.html‎). The multiple aligned sequences were used as an input file for constructing each tree. An unrooted linear Neighbor joining (NJ) tree was plotted with an option of 10,000 bootstrap values. Missing gaps were excluded from the analysis. Gene tree using Maximum Parsimony, Minimum Evolution and UPGMA method were also drawn to verify consistency.

### Allelic expression analysis

Based on the sequence and genotypic screening analysis through marker study conducted with pTA248 marker [38] for *Xa21* and *xa5* functional marker [39], four alleles each of the genes *Xa21* and *xa5* were selected for expression analysis. Selected alleles included IRBB21 (*Xa21*) and IRBB5 (*xa5*) as resistant controls and IR24 as a susceptible control for both genes. The *Xa21* test alleles were from two accessions of *O*. *longistaminata* and the *xa5* test alleles were from Kalamekri and AC32753 (*O*. *sativa*). Expression analysis of *Xa26* alleles were not performed as *Xa26* gene being reported as constitutively expressed gene [40]. The rice plants were grown in glass house with adequate water and nutrients. The day and night temperature of the glass house was maintained at 28°C. Plants at booting stage were then inoculated with *Xoo* strain DX011 (which was incompatible with rice genotypes possessing *Xa21* and *xa5*) in triplicates by leaf clipping method [26]. The control plants were treated in a similar manner with sterile water instead of *Xoo* inoculums. Leaf samples for RNA isolation were collected at different time intervals between 0 and 24 h. Total RNA was extracted with 100 mg leaf samples using an RNA isolation kit (Genetix, India) according to the manufacturer’s instructions, from triplicate samples. Extracted RNA was treated with DNase to remove genomic DNA impurities. Equal amounts of RNA (1–2 μg) were taken from each sample and cDNA was synthesized using Superscript III cDNA synthesis kit (Invitrogen, USA) following manufacturer’s instructions. For the *Xa21* alleles, semi quantitative RT-PCR was performed. A 30 μL reaction mixture consist of 100 ng cDNA, 10X PCR buffer (10 mM Tris-HCl, pH 8.0, 1.5 mM MgCl_2_), 4 pmole of each primer, 0.2mM of each dNTPs and 0.6 unit (2U/μL) of *Taq* polymerase (Invitrogen, USA). Initial denaturation was set for 4 min at 95°C with 26 cycles of amplification with 45 s DNA denaturation at 94°C, 30 s annealing at 60°C and extension at 72°C for 1 min. Primers for *xa5* and *Xa21* were designed from the exonic junction (S1 Table). Actin primer (Forward 5' GAGTATGATGAGTCGGGTCCAG 3’ and Reverse 5’ ACACCAACAATCCCAAACAGAG 3’) was used as reference gene for both semi-quantitative and quantitative RT-PCR (qRT-PCR) [41]. For qRT-PCR, a master mix containing 2X Sybr Select Master Mix (Invitrogen, USA), 150–400 nM forward and reverse primers, and 100 ng of cDNA was used as a template. The PCR program was set for UDG activation at 50°C for 2 min, AmpliTaq DNA Polymerase UP activation at 95°C for 2 min, and denaturation at 95°C for 15 s. Primer annealing was performed at the melting temperature of the primer for 15 s and extension at 72°C for 1 min. The cycle was repeated for 40 times from denaturation step. The real time data were analyzed by relative quantification methods and was calculated using the following formula:

$$\begin{array}{l} \delta \delta C_{T}={\left(\delta C_{T}{,}_{Target}-\delta C_{T}{,}_{Reference}\right)}_{Time\;x}-{\left(\delta C_{T}{,}_{Target}-\delta C_{T}{,}_{Reference}\right)}_{Time\;0}\\ \delta C_{T,\;Target}=\;C_{T,\;Control}-\;C_{T,\;Treatment,}\delta C_{T\;Reference}=\;C_{T,\;Control}-\;C_{T,\;Treatment} \end{array}$$

Fold changes in expression was expressed as $2^{-\delta \delta C}{}_{T}$ [42]_._ Statistical significance of the data was analyzed using two way ANOVA and Bonferroni post test.

## Results

### Nucleotide Polymorphism analysis of *Xa21*, *Xa26* and *xa5* loci

#### Sequence analysis of *Xa21* alleles

The total ORF of *Xa21* comprising of 3,921 nucleotides including one intron of 843 base pairs was sequenced from 18 accessions. Allelic sequences ranged between 3,828 to 3,921 nucleotides and the length of the total aligned sequences was 3,997 base pairs. The percentage identity of the nucleotide sequences of the alleles ranged between 95–99% in comparison to IRBB21. There were a total of 196 mutations, 191 segregating sites, 113 parsimony informative sites, 64 InDel events and 78 singleton variable sites (rare kind of polymorphism, which is present once in the sample) within the entire sequenced region. The overall average number of nucleotide difference (K) was found to be 41.977 (Table 2). There were a total of 136 SNPs in exonic regions and 40 SNPs in the intron. The frequency of SNP was one SNP per 22.7 bp for the entire sequence, one SNP per 23 bp for the exonic region and one SNP per 21.5 bp for the intron. The SNP frequency was higher in *O*. *sativa* with one SNP per 51.9 bp and one SNP per 55.25 bp in *O*. *nivara*. The average frequency of InDel polymorphism was one InDel site for every 62.4 bp. Maximum InDel was observed in the first exon and least in the last exon. The frequency of InDel was highest in the intron followed by exon 1 and least in exon 2. Nucleotide substitution was observed throughout *Xa21* but the highest nucleotide diversity was seen within the first 500 bp of the coding region. The nucleotide diversity π of the total sequence was 0.01112 and the intronic region had higher nucleotide diversity (π = 0.01328; *Ө*_*w*_ = 0.01500) than the coding region (π = 0.01055; *Ө*_*w*_ = 0.01433). Within the coding region, exon 1 (π = 0.01083) had substantially higher diversity than exon 2 (π = 0.00874). *O*. *nivara* exhibited slightly higher sequence variation (π = 0.00782; *Ө*_*w*_ = 0.00846) compared to *O*. *sativa* (π = 0.00688; *Ө*_*w*_ = 0.00782). Nucleotide diversity was higher than InDel diversity (0.00238). All alleles, except OLD and OL1, showed an equal level of polymorphism (S1 Fig.). Frequency of ‘A’ to ‘G’ transition was the most frequent polymorphism and overall transition bias of the alleles was 1.221.

**Table 2:** Summary statistics of nucleotide diversity analysis of *Xa21*, *Xa26* and *xa5* genes.

Gene		Total sites	S	Π	*Ө*_*w*_	Fu and Li’s D	Tajima’s D	K	Parsimony informative site	Singleton variable sites
*Xa21*	Entire gene	3997	191	0.0111	0.0144	−0.72	−1.05476	41.977	113	78
	Coding region	3141	150	0.0108	0.0143	−0.900	−1.183	31.59	84	66
	*O*. *nivara*	3997	88	0.0078	0.0084	−0.298	−0.387	29.94	45	43
	*O*. *sativa*	3997	73	0.0068	0.0078	−0.754	−0.764	26.23	23	50
*Xa26*	Entire gene	3987	304	0.0195	0.0274	−1.709	−1.297	63.072	122	182
	Coding region	3528	260	0.0168	0.0233	−1.566	−1.2754	54.556	149	111
	*O*. *nivara*	3987	190	0.0221	0.0227	−0.8222	−0.1833	75.52	98	92
	*O*. *sativa*	3987	248	0.0213	0.027	−1.11	−1.18	72.25	169	79
*xa5*	Entire gene	6395	310	0.0132	0.0153	−0.1903	−0.6719	78.9	205	105
	*O*. *nivara*	6395	154	0.011	0.0102	0.0042	0.2033	67.57	84	70
	*O*. *sativa*	6395	102	0.0073	0.0064	0.4803	0.7487	44.75	70	32

S = No. of polymorphic sites, K = Average nucleotide difference, π = Nucleotide diversity, Ө_w_ = No. of segregating sites

Divergence at the synonymous site (π_syn_ = 0.01439) was higher than the non-synonymous site (π_non_ = 0.00876). Most of the polymorphic sites in the coding region resulted in silent substitution, both conservative and non-conservative amino acid changes along with internal stop codons in all 18 alleles. We observed that the value of K_s_ was greater than K_a_ leading to the ratio of K_a_/K_s_ < 1, indicating the alleles were under the process of purifying selection with respect to IRBB21 (Table 3). Tajima’s test showed no significant difference between π and *Ө*, thus, showing consistency with the neutral theory [43]. The Tajima’s D value was estimated to be -1.05476 (*P* > 0.10,). Similarly, separate Tajima’s D test for coding, non-coding and intraspecies also showed negative and non-significant departure from neutrality. Linkage Disequilibrium was significant for both *O*. *nivara* (R^2^ > 0.02) and *O*. *sativa* (R^2^ > 0.03) (0.001< P < 0.01). LD plots of R^2^ values as a function of pairwise distance between polymorphic sites revealed slight decay of the analyzed loci within 3,500 bp in *O*. *sativa* and 4,000 bp in *O*. *nivara* (S2 Fig.). Fisher’s exact test and Chi-square test provided the number of significant pairwise comparisons. While determining the recombination sites of this locus, a minimum of seven Rm events were detected considering only the coding region.

**Table 3:** Summary statistics of different types of mutations present in *Xa21*, *Xa26* and *xa5* alleles.

Gene		SNPs	InDels	π_s_	π_a_	π_a_/ π_s_ or Ka/Ks (Jukes & Cantor)
*Xa21*	Entire gene	165	64	-	-	-
	Coding region	122	45	0.01439	0.00876	0.6087
	*O*. *nivara*	84	38	0.00965	0.00679	0.7036
	*O*. *sativa*	49	24	0.00890	0.00569	0.6393
*Xa26*	Entire gene	257	61	-	-	-
	Coding region	253	38	0.02299	0.01507	0.6555
	*O*. *nivara*	119	38	0.02483	0.01760	0.7088
	*O*. *sativa*	176	37	0.02490	0.01683	0.6759
*xa5*	Entire gene	232	87	-	-	-
	Coding region	9	0	0.00710	0.00566	0.7971
	*O*. *nivara*	98	45	0.00411	0.00193	0.4695
	*O*. *sativa*	92	33	0.00384	0.00941	2.45

SNPs = Single nucleotide polymorphism, InDels = Insertion Deletion, π_s_ = average number of nucleotide diversity at synonymous sites, π_a_ = average number of nucleotide diversity at non synonymous sites.

#### *Xa21* gene tree and molecular evolution

**Fig 1:**
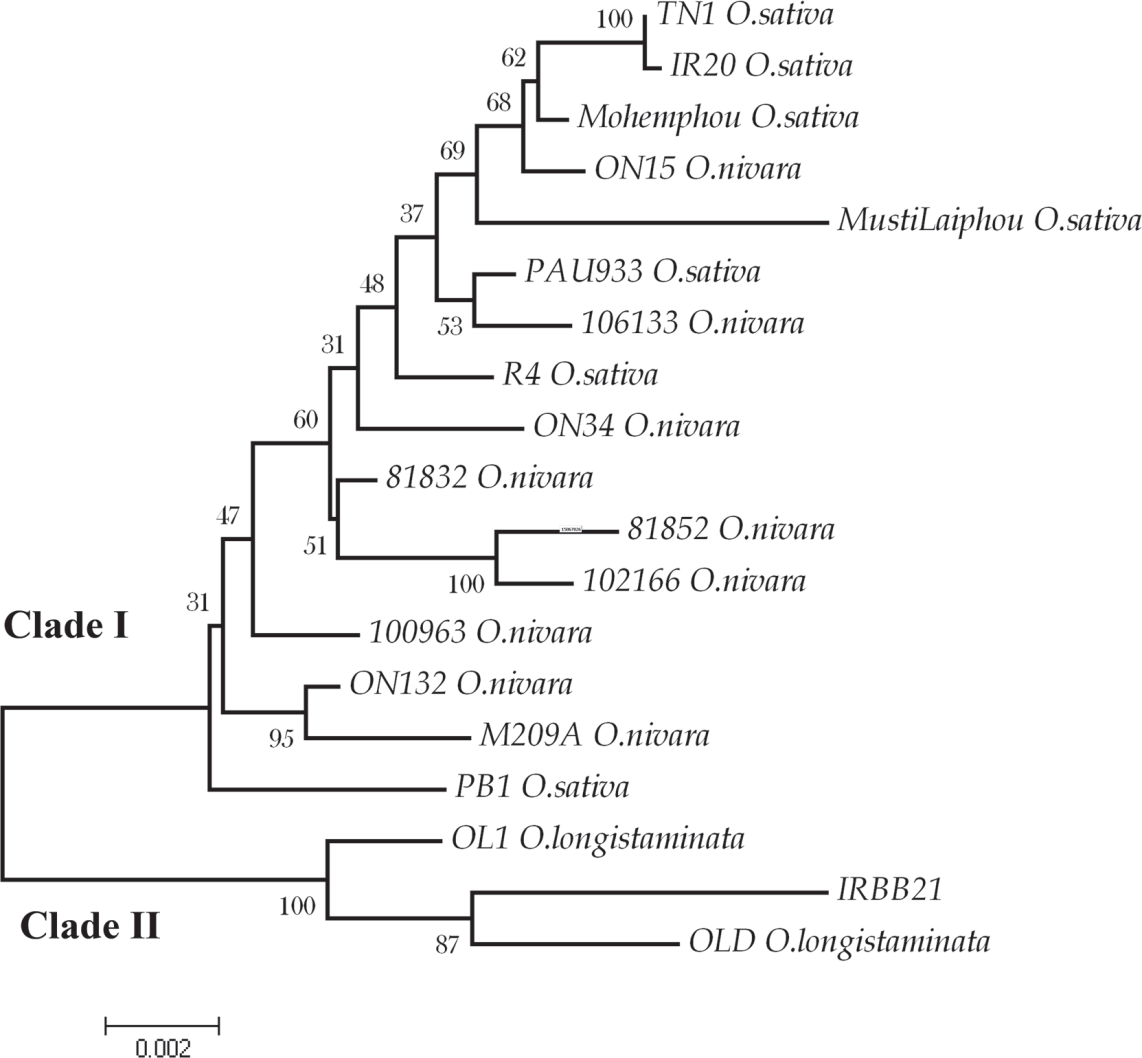
A Neighbor-Joining plot showing the evolutionary relationship between *Xa21* alleles. The tree was constructed using 10,000 bootstrap values. The scale of the tree with branch lengths is indicated at the bottom. There are two major clades, clade I and II. The IRBB21 related alleles were clustered together in clade II. Each allele is indicated with the accession name followed by species name. Phylogenetic analyses were conducted in MEGA4 (Tamura et al. 2007).

Nucleotide sequences were used to deduce neighbor joining and parsimony trees. Two major clades, I and II, were present which were supported by high bootstrap values (Fig. 1). Clade I comprised two minor clades. IRBB21 and two alleles from *O*. *longistaminata* were clustered separately in clade II, showing that these alleles were highly distinct from the remaining alleles of different species (Fig. 1). These three alleles emerged together in NJ, ME, MP and also UPGMA. Between clade I and II, there were 33 fixed differences across the entire sequence. Clade II showed slightly higher within clade diversity (π = 0.00946) than those of clade I (π = 0.00772). There were 55 singletons and 0 parsimony sites in clade II and 61 singletons and 60 parsimony informative sites among alleles in clade I. Highest divergence among the alleles was observed between IRBB21 related and remaining alleles. The intra specific divergence was quite low compared to inter specific divergence; however the divergence between alleles of *O*. *sativa* and *O*. *nivara* individuals were minimal (S3 Fig.).

#### Allelic expression analysis of *Xa21*

**Fig 2:**
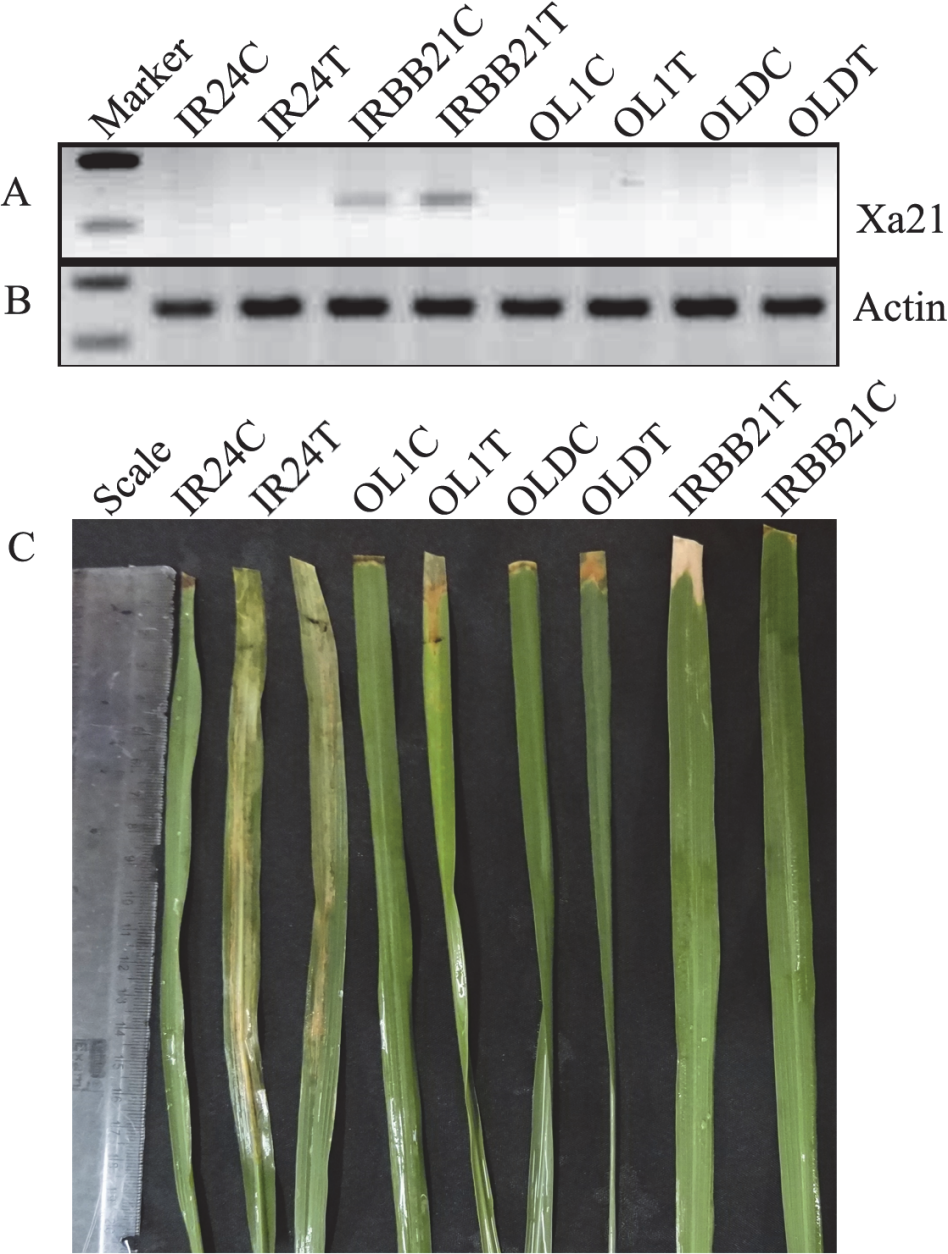
Semi-quantitative PCR analysis of *Xa21* alleles in control and treated samples of IR24, IRBB21, OL1 and OLD. (a) Expression of *Xa21* gene in IRBB21 control and infected with *Xoo*. *Xa21* expression is absent in remaining samples. (b) Expression pattern of reference gene rice actin in the samples. (c) Phenotypic response of the genotypes after *Xoo* infection. Leaves with lesion length more than 5 cm are susceptible. IR24 and IRBB21 was used as susceptible and resistance controls, respectively. Control (C), Treated/Infected (T).

In order to confirm the functional significance of alleles identical to resistant *Xa21* (IRBB21), we checked the expression of *Xa21* alleles in two accessions of *O*. *longistaminata* (OL1 and OLD). However, these two tested alleles were not expressed or detected before or after infection. We could see the up regulation of this gene only in IRBB21 after infection as shown by Semi-QRT-PCR (Fig. 2).

### Sequence analysis of *Xa26* alleles

Alleles of *Xa26* were isolated from 14 rice accessions of *O*. *sativa* and *O*. *nivara*. Complete sequence analysis was performed with the alleles from IR24, IRBB3, Minghui 63 and Zhachanglong (*O*. *sativa*). Sequence polymorphism was detected across 3879 bp of sequence covering 3315 bp in exonic regions, 106 bp in intronic regions and 458 bp in the 3’UTR. The total alignment length was 3987 bp. The percentage sequence similarity among alleles ranged between 95–99%. IRBB3 showed least sequence similarity with IC203740 and IR24 of 97% and 96%, respectively.

Nucleotide sequence polymorphisms were analyzed across 18 accessions of different species on the *Xa26* locus. There were a total of 304 polymorphic (segregating sites) and 312 mutations with an estimated average nucleotide divergence (K) of 63.072. Among them, 182 sites belonged to singleton variable and 122 parsimony informative sites. There were 16 haplotypes among 18 alleles and the haplotype diversity, Hd = 0.98 ± 0.028. The overall nucleotide diversity was π = 0.01958 and the Watterson estimator ‘*Ө*_*w*_’ = 0.02744 (Table 2). The highest nucleotide diversity was observed in the 3’ UTR followed by coding region (π = 0.01684). As many as 61 InDel events and 315 nucleotide substitutions were present. Frequency of SNPs was more than the InDels being one SNP per 24.91 bp and one InDel per 65.36 bp. The average InDel length was longer in the coding region (8.2271) as compared to the entire gene (2.15) and its InDel diversity (π_i_ = 0.0012) was least different from that of coding region (π_i_ = 0.00091). The longest InDel was found in two *O*. *nivara* accessions (ON132 and 210223). Among alleles, the highest number of polymorphisms was found between IR24 and IRBB3. Many of the SNPs and InDels occurred in the exons, which resulted in stop codons and premature termination of coding frame. Pattern of nucleotide substitution was estimated by the maximum composite likelihood method where the overall transition bias ‘R’ = 1.421, the highest among the four genes. The ‘G’ to ‘A’ transition was the most favored transition and the transition and transversion rate was almost the same in all alleles when compared individually with IRBB3.

Similarly, as observed for *Xa21* alleles, the divergence at silent site (π_syn_ = 0.02299) was higher than the non-synonymous site (π_a_ = 0.01507). When individual alleles were compared with IRBB3/Minghui 63, the total number of synonymous substitutions were more than the non-synonymous changes resulting in Ka/Ks < 0, which illustrates that the alleles are undergoing purifying selection with reference to *Xa26* of IRBB3 (Table 3). For the neutrality test, the Ө value was greater than π giving negative Tajima’s D (-1.297, p > 0.10) (Table 2). The difference was statistically not significant, which illustrates an absence of significant selection of this gene in our study population. Out of the 14 alleles isolated, only two alleles (M209A and 106133) showed an intact ORF giving a full length protein, whereas internal stop codons were found in the sequences of the remaining alleles. Amino acid substitutions were found both in LRR and kinase domain.

As in the case of *Xa21* alleles, interspecific nucleotide diversity was slightly higher in *O*. *nivara* (π = 0.02216) than *O*. *sativa* (π = 0.02139). However intra specifically, *O*. *sativa* (254) had more mutations than *O*. *nivara* (191). Both species showed negative Tajima’s D with non-significant departure from neutrality. The linkage disequilibrium was found to be significant for both *O*. *sativa* (R^2^≈ 0.54) and *O*. *nivara* (R^2^ = 0.7). Decay in LD was also observed in both the species when R^2^ was plotted against pairwise distance between polymorphic sites (S4 Fig.).

#### Molecular evolution tree of *Xa26*

**Fig 3:**
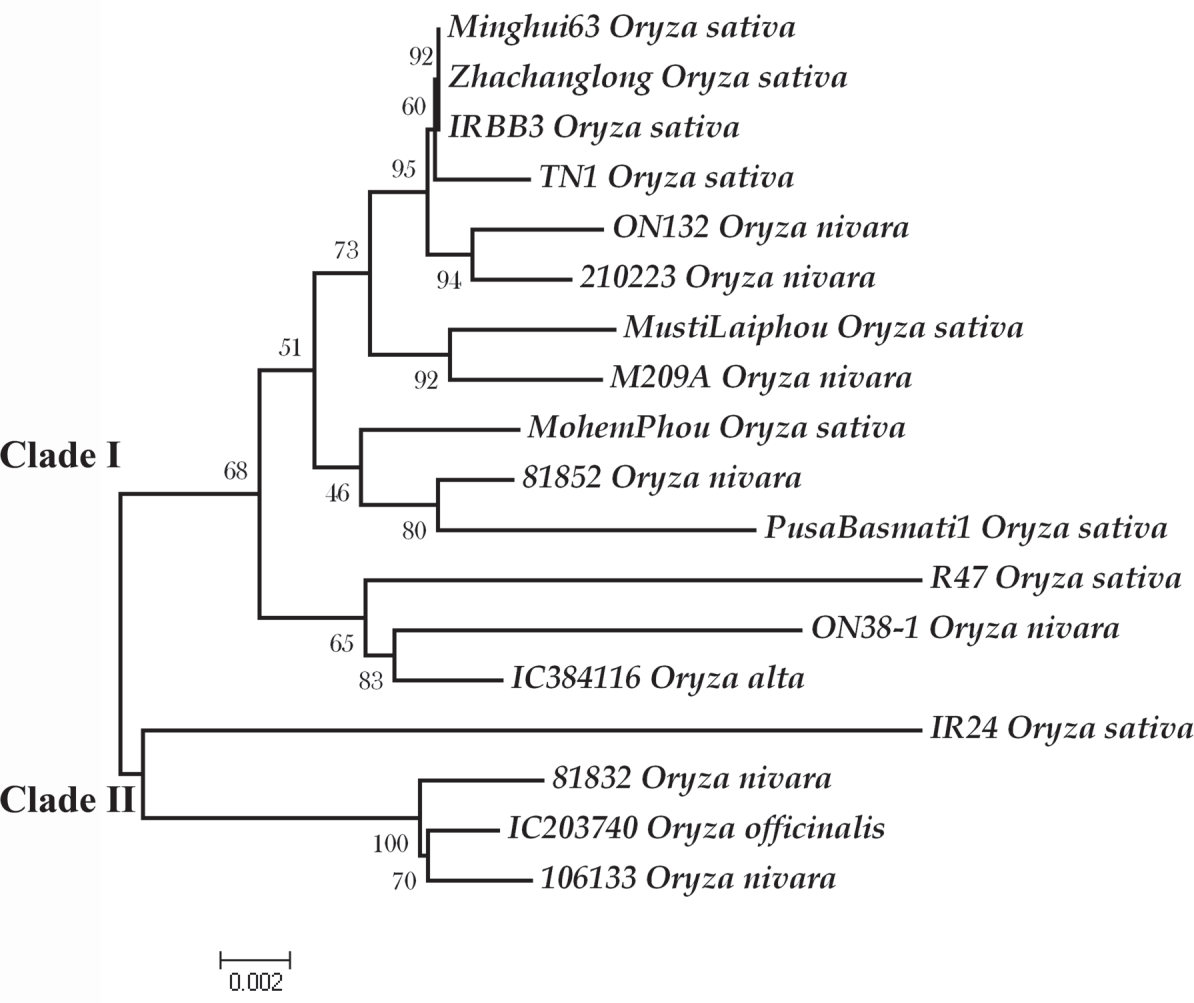
Neighbor joining gene tree depicting the relationship of all studied individuals at *Xa26* locus. The tree was inferred from 10,000 replicates bootstrap values to represent the evolutionary history of the alleles. The highest bootstrap value of each branch is indicated correspondingly. The scale of branch length is indicated at the bottom. Each allele is indicated with the accession name followed by species name. Phylogenetic analyses were conducted in MEGA4 (Tamura et al. 2007).

A dendrogram depicting the relationship and divergence of 18 *Xa26* alleles was plotted using Maximum Parsimony and NJ. The plot formed two major clades supported by strong bootstrap values. Clade I had resistant alleles from IRBB3, Minghui 63 and Zhachanglong while two susceptible alleles from *O*. *sativa* (PB1 and IR24) were clustered in clade II. The cluster representing the remaining alleles from *O*. *nivara*, *O*. *sativa*, and *O*. *officinalis* were not distinguished at the species level (Fig. 3). Intermixing of genetic components was observed, which may be due to outcrossing.

### Sequence analysis of *xa5* alleles

*xa5* alleles were isolated from 15 accessions, out of which alleles from two *O*. *sativa* accessions were similar to recessive *xa5* and remaining alleles were similar to dominant *Xa5*. Comparative analysis of the alleles with respect to IRBB5 showed an average sequence identity in the range of 97–99%. The total alignment length was 6395 sites and size of the alleles ranged between 6140 and 6264 bp. The comparative sequence analysis showed 310 polymorphic sites (out of 316 total mutations), 105 singleton variable sites and 205 parsimony informative sites. There were a total of 16 haplotypes (Hd = 0.993 ± 0.023) and 87 InDel events. The frequency of SNPs and InDels were higher in the intronic and the non-coding region compared to the exonic region. The mean frequency of InDel was present at every 73.5 bp and that of SNP was one SNP per 27.56 bp. As observed in previous studies [13], transition/transversion bias was present with R = 1.066, where ‘G’ to ‘A’ and ‘C’ to ‘T’ transitions were most prevalent. In the exonic region, we observed significant SNPs consisting of both synonymous and non-synonymous substitutions. Apart from the type of substitution reported by Iyer et al. [24] we found 9 additional SNPs in the coding region. Among these SNPs, 7 resulted in amino acid substitution (S5 Fig.) and included an ‘S’ to ‘A’ substitution in AC32753 (*O*. *sativa*) and a ‘Q’ to ‘L’ in PB1 (*O*. *sativa*) which were non conservative amino acid substitutions. The frequency of SNP was higher in *O*. *nivara* accessions (one SNP per 63.31 bp) than *O*. *sativa* (one SNP per 70.27 bp). We also observed 39 fixed differences between *O*. *longistaminata* and remaining accessions.

The overall diversity analysis showed a mean nucleotide diversity of π = 0.01324, *Ө*_*w*_ = 0.01539 and an average nucleotide difference of K = 78.904. Maximum diversity was observed in the second intron. Intraspecific polymorphisms were found to be higher in *O*. *nivara* (π = 0.01106, *Ө*_*w*_ = 0.01029) than *O*. *sativa* (π = 0.00733, *Ө*_*w*_ = 0.00644) (Table 2). Ka/Ks values for each allele were determined in comparison to *xa5* (IRBB5) sequence. Ka/Ks were greater than one for PB1, TN1 (*O*. *sativa)*, 81832 and 81852 (*O*. *nivara)*. The selection was neutral for AC32753 and Kalamekri while, remaining alleles were undergoing purifying selection with respect to IRBB5. We observed a similar trend in all the genes where Tajima’s test showed no significant difference between π and *Ө*, which is consistent with the neutral theory [35]. Though, some individual alleles showed Ka/Ks > 1, the average Tajima’s D was estimated to be -0.67191 (*P* > 0.10). Similarly, separate Tajima’s D test for intraspecies variation was negative and did not depart from neutrality.

Linkage Disequilibrium was significant for both *O*. *nivara* (R^2^ > 0.75, Panel A in S6 Fig.) and *O*. *sativa* (R^2^ > 0.55, Panel B in S6 Fig.) (0.001< P < 0.01). The LD plot of R^2^ values as a function of pairwise distance between polymorphic sites revealed slight decay of the analyzed loci within 4000 bp in *O*. *nivara*. The pairwise comparison was significant as determined by Fisher’s exact test and Chi-square test. While determining the recombination sites at this locus, zero Rm events were detected considering only the coding region.

#### *xa5* gene tree and molecular evolution

**Fig 4:**
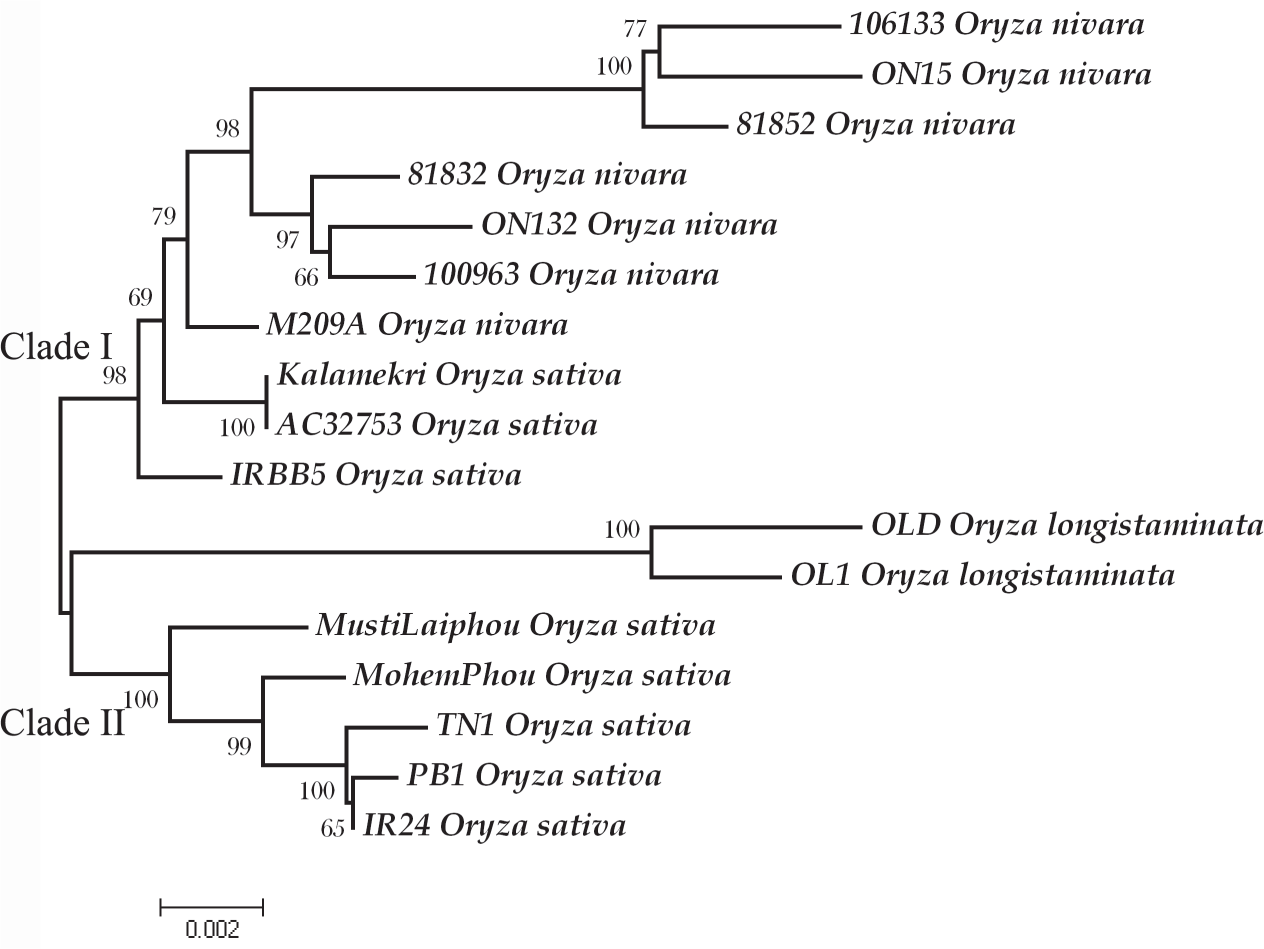
A neighbor joining gene tree of all studied individuals at *xa5* locus. The tree was constructed with 10,000 bootstrap values. The highest bootstrap value is indicated on each branch. The scale of branch length is indicated at the bottom. Each allele is indicated with the accession name followed by species name. The alleles are clustered separately at the species level, while the *xa5* alleles (resistant and recessive form) IRBB5, AC32753 and Kalamekri show more relatedness to *O*. *nivara*.

An unrooted phylogenetic tree (NJ plot) depicting genetic relatedness among the alleles was plotted using Mega 4. The entire sequence comprising 5’UTR, coding, non-coding and 3’UTR were considered in plotting the tree. Two major clades, supported with high bootstrap values, were present in the plot. Resistant alleles were found to be closer to alleles from *O*. *nivara* accessions than the susceptible alleles from *O*. *sativa* accessions. IRBB5 was found clustered in clade I along with other *O*. *nivara* accessions (Fig. 4). A similar pattern of clade formation was also observed with ME, MP and UPGMA tree. Within clades, diversity was found to be higher in clade II (π = 0.01288) than clade I (π = 0.01040). There were 66 singleton variable sites, 109 parsimony informative sites in clade I and 76 SVS and 114 PIS in clade II. In disparity index analysis, the highest composite distance was found between 81852 (*O*. *nivara*) and OLD (*O*. *longistaminata*) (S7 Fig.).

#### Allelic expression analysis of *xa5*

**Fig 5:**
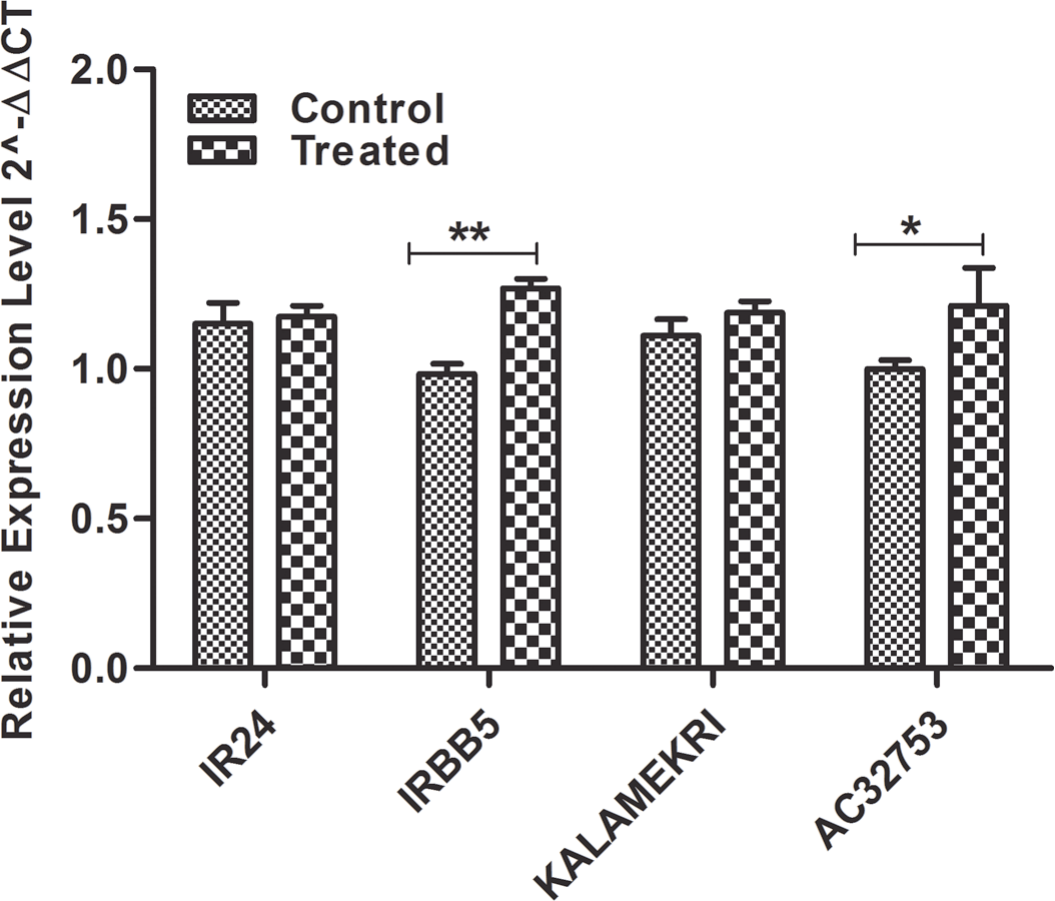
This graph depicts the expression pattern of *xa5* alleles in control and treated samples of IR24, IRBB5, AC32753 and Kalamekri. ** P value significant with P < 0.01; * P value < 0.05 significant confirmed through two way ANOVA Bonferroni post test.

Expression analysis of *xa5* alleles identified from accessions AC32753 and Kalamekri were tested using IRBB5 and IR24 as resistant and susceptible, respectively. We were interested to see if any of the SNPs in the alleles other than the previous report had any influence in its expression or not. Only basal level of expression of these alleles were observed in all the tested samples (Fig. 5) which suggests the level of expression of this gene is not related to its resistance towards the pathogen.

## Discussion

*Functional variations of genes which lead to different phenotypes are being influenced by polymorphisms such as* SNPs and InDels of single nucleotide or large DNA fragments. As a natural phenomenon, different allelic forms of *R* genes are maintained in a population so as to protect plants against evolving pathogens [2, 14]. The high-end, low-cost next generation sequencing technologies in combination with vast genetic resources available in rice, gives us an opportunity to discover potential resistance gene and trace their evolutionary pattern. In this study, we have analyzed the sequence polymorphisms of three major bacterial blight resistance genes *xa5*, *Xa21* and *Xa26* in order to identify novel alleles. This may help to uncover genetic variants of these genes in wild and cultivated species.

Among the resistance genes of bacterial blight disease, *Xa21* is one of the most commercially exploited and studied gene. It has been reported to confer broad spectrum resistance to diverse *Xoo* strains across the world, yet there are reports on the susceptibility of *Xa21* to pathotypes of Indian and South Asian origin [44–48], which may be due to mutation and evolution of these isolates. Discovery of candidate disease resistance alleles and analysis of novel variant alleles of *Xa21* will help in combating new and more virulent strains of pathogens. *Xa26* is another receptor kinase gene, which is structurally similar to *Xa21*. Phenotypically, even though IRBB3 (which has *Xa3/Xa26*) was susceptible to the five virulent isolates used in our study, we were interested in understanding its molecular diversity statistics, hence this gene was considered for analysis. *xa5*, on the other hand is a recessive resistance gene. Finding any sequence variation which influences the activity of this gene other than the previously reported forms of mutations would be interesting.

Nucleotide diversities of *Xa21*, *Xa26* and *xa5* genes were found to be comparatively less as compared to reported *R* genes of Arabidopsis and other crop plants [49–51]. At the species level, we found a slightly higher diversity in *O*. *nivara* than the cultivated *O*. *sativa*. We also observed a decrease in diversity among *O*. *sativa*, which may be due to the domestication bottleneck [52–54]. Under natural conditions, introns evolve more rapidly than exons [55], likewise sequence polymorphisms were higher in the intron and non-coding region than coding region, which is often seen in most of the genes studied. Haldane [51] stated that high polymorphism is expected at the locus involved in pathogen recognition; likewise, elevated levels of nucleotide and amino acid polymorphisms were observed at *Xa21* and *Xa26* loci, which also have an LRR domain. LRR are known to be involved in pathogen recognition. Numerous substitutions and InDels resulted in amino acid polymorphisms resulting in non-functional genes, including two alleles of *Xa21* from *O*. *longistaminata* that were due to a premature termination codon, which were confirmed by expression analysis.

Universally, transition bias is prevalent across different kingdom, genera and species and has been observed to be a common phenomenon during the course of evolution. The alleles studied herein showed more transitions from ‘G’ to ‘A’ and ‘C’ to ‘T’ than vice versa. A similar feature has been observed in prokaryotes [56], nematodes [57], Drosophila, mammals [58] and plants [59]. High rates of ‘C’ to ‘T’ transitions may occur due to methylation of cytosine, which increases the probability of this form of substitution [60]. It was difficult to determine the ancestral root of the alleles from the phylogenetic tree as the trees were unrooted. Among *Xa21* alleles, OL1 and OLD showed maximum homology with IRBB21. This finding defined their genetic relatedness and similar origin, as *Xa21* in IRBB21 was also derived from *O*. *longistaminata*. The presence of two divergent groups and distinctness of IRBB21 related alleles from remaining alleles were evident from the divergence table and two separate major clades supported with high bootstrap values in the phylogram (Fig. 1). For *xa5* and *Xa26* genes, a clear differentiation among alleles at the species or phenotypic level was difficult to determine from the gene tree. For a comprehensive study to elucidate evolutionary relationship of the alleles, it is vital to increase the number of natural populations of *O*. *sativa* and *O*. *nivara*.

Tajima’s D statistics signify the nature of selection at a locus. Natural selection was indicated in the case of rice blast resistance genes in the *O*. *rufipogon* population and *R* genes in other plants with significant Tajima’s D value. However, the resistance genes analyzed in this study failed to show a significant Tajima’s D, which suggest the absence of natural selection at all the three loci. However, the possibility of selection at these loci cannot be precluded as the samples under study were small in size and randomly selected from a natural population [13]. A large number of polymorphisms among the alleles leading to amino acid substitution and premature termination of protein translation were also observed. In addition, the negative Tajima’s D value, though not significant, shows an excess of rare variants and indicates the likelihood of selection as reported in the case of *RPP13* [51] and *Pto* [2]. Alternatively, relaxed selection pressure or the genes under consideration might be evolving neutrally resulting in the gene being depleted from the population through deletion, frame-shift or nonsense mutation [51].

LD was measured by plotting R^2^ as a function of pairwise distance between the SNPs. The LD value was found to be the same as that of other plants reported. A locus specific selection leads to an increase in the LD. Higher R^2^ was observed in *O*. *sativa* than *O*. *nivara* and the extent of LD was higher in *O*. *nivara* in the case of *Xa21* alleles. In contrast, a much higher value of R^2^ and rapid decay in LD was observed in *O*. *nivara* than *O*. *sativa* for *Xa26* and *xa5* alleles. This decay in LD may be because of high recombination rate, which mainly happens in the cross pollinated plants. Recombination and cross pollination influences decay of LD.

Though, OL1 and OLD showed the presence of an identical allele of *Xa21*, functionally, these alleles failed to show any expression after pathogen infection. Phylogenetically and based on sequence analysis, these two alleles were nearest to IRBB21 (resistant allele), however, they were non-functional due to nonsense mutations in the coding region. These two alleles were not expressed, even after infection with a pathogen while IRBB21 was observed to be expressed (Fig. 2). Since, OL1 and OLD were found to be resistant to BB, resistance in these two accessions might be contributed by loci other than *Xa21*. Further studies with segregating population will confirm this. Looking for *Xa21* alleles in more *O*. *longistaminata* accessions and other wild species may help in finding the desired form of the allele. As in the case of *xa5* alleles, IRBB5, susceptible IR24, and the remaining tested alleles AC32753 and Kalamekri showed almost equal level of expression both under infected and non-infected state. This indicates the level of expression of the alleles might not influence its function, however, their ability or non-ability to bind DNA polymerase and to manipulate the induction of susceptibility genes may determine their role in resistance [61]. The functional significance of amino acid substitutions in AC32753, 81832 (*O*. *nivara*) and 81852 (*O*. *nivara*) may be elucidated however, structural amino acid substitutions in these accessions occurred in the loop region. Hence, functional changes in these alleles with respect to resistance/susceptibility due to these mutations are least expected. Phenotypically, AC32753 and Kalamekri were resistant to the disease and these two alleles may be employed for gene pyramiding into elite cultivars, after genetically establishing that resistance in these two accessions is controlled by novel alleles of *xa5* through analysis of segregating populations.

In summary, we have analyzed the genetic diversities of three major BB resistance genes in rice. This analysis is the first of its kind for BB related genes after *Xa27* and may also help in molecular evolutionary studies and mining useful alleles for crop improvement. The major contribution of these variations among alleles on BB management has yet to be analyzed and their potential impact ascertained. Including more individuals from natural populations in the analysis after phenotypic screening with pathogens possessing specific *Avr* genes will help in determining the co-evolutionary relationship between host resistance and pathogen. A comparative study on susceptible and resistance accessions which define their distinct reaction towards pathogens may help in developing effective strategies for managing the infectious plant disease. Some of the accessions identified in this study which showed high resistance to BB will be used as donor lines for BB resistance genes to BB susceptible elite cultivars. The alleles identified in this study will also be transferred to elite susceptible varieties and further confirmed their functional significance.

## Supporting Information

S1 Table List of primers used for isolation of alleles and expression analysis. (DOCX)

S1 Fig Graph depicting different types of polymorphisms found at *Xa21* locus among different accessions. Blue, red and green colors indicate InDels, SNPs and total number of polymorphic sites, respectively. Different accessions are shown in X-axis and number of polymorphisms on Y- axis.
(TIF)

S2 Fig Pattern of Linkage disequilibrium among *Xa21* alleles (A) *O*. *nivara* (B) *O*. *sativa*. Decay of LD ‘R^2^’ as a function of distance between pairs of polymorphic sites in *Xa21* alleles. The Black line depicts the expected decline of LD against distance based on the equation given by HILL and WEIR (1988).
(TIF)

S3 Fig Estimates of base composition bias difference between sequences of *Xa21* alleles. The difference in base composition bias per site is shown in each column. Left column indicate the alleles and the numbering columns indicate the divergence rate corresponding to the numbered rows.
(TIFF)

S4 Fig Pattern of Linkage disequilibrium among *Xa26* alleles (A) *O*. *nivara* (B) *O*. *sativa*. Decay of LD ‘R^2^’ as a function of distance between pairs of polymorphic sites in *Xa26* alleles. The Black line depicts the expected decline of LD against distance based on the equation given by HILL and WEIR (1988).
(TIF)

S5 Fig (A) Multiple alignment of coding region of xa5 alleles. Substitutions in the CDS are represented with different colors. (B) Output of multiple alignment for the predicted amino acid sequences of *xa5* alleles. The rice genotypes are indicated in left column. The numbers on the top of the sequences indicate the position of amino acids. Different colors in amino acid shows non synonymous changes. Alignment was performed using Multalin program.
(TIF)

S6 Fig Pattern of Linkage disequilibrium among *xa5* alleles (A) *O*. *nivara* (B) *O*. *sativa*. Decay of LD ‘R^2^’ on X-axis as a function of distance between pairs of polymorphic sites in *xa5* alleles and nucleotide distance on Y-axis. The Black line depicts the expected decline of LD against distance based on the equation given by HILL and WEIR (1988).
(TIF)

S7 Fig Estimates of base composition bias difference between sequences of *xa5* alleles. The difference in base composition bias per site is shown in each column. Left column indicate the alleles and the numbering columns indicate the divergence rate corresponding to the numbered rows.
(TIF)
