# Supplementary material for: Nucleotide Diversity Analysis of Three Major Bacterial Blight Resistance Genes in Rice
Source: PLoS One. 2015 Mar 25;10(3):e0120186. doi: 10.1371/journal.pone.0120186 (PMC4373814; doi:10.1371/journal.pone.0120186)
Supplement: S1 Table — (DOCX) [file pone.0120186.s001.docx]

**S1 Table.** List of primers used for isolation of alleles and expression analysis

| Gene | Gene specific Primer | Sequence 5’ to 3’ | Tm (ºC) | Product size (bp) |
| --- | --- | --- | --- | --- |
| *xa5* | Xa5.1F | AGCAGCATTTCCAAGAGTGG | 54 | 1030 |
|  | Xa5.1R | TAC GTG TCG AAC GTG AAT GG |  |  |
|  | Xa5.2F | CTTTTCCGTACACACCTTGC | 54 | 1234 |
|  | Xa5.2R | ACA ACC GAG TGA CCC AAA GG |  |  |
|  | Xa5.3F | CGAAATTCTGTGTGGGTTGC | 53 | 1130 |
|  | Xa5.3R | TTA GCT CAG CAG GGA GAA GC |  |  |
|  | Xa5.4F | TGCTACCGGAGACTAGAAAGG | 62 | 1200 |
|  | Xa5.4R | AGG TGT GCC GTA TTT AGA AGC |  |  |
|  | Xa5.5F | ACGGCACACCTTTATCATCC | 54 | 690 |
|  | Xa5.5R | CCA GTT CTC CTG CAT TTT CG |  |  |
|  | Xa5.6F | CGAAAATGCAGGAGAACTGG | 54 | 950 |
|  | Xa5.6R | GCC CTG AGA AAA GAA CAT GC |  |  |
|  | Xa5.7F | GACGCAATGTCGAACAGACC | 60 | 750 |
|  | Xa5.7R | GCC AAA TGT TGC TAG GGT TG |  |  |
|  | Primer for RT |  |  |  |
|  | X5RTF1 | GAGCAAGGTTTCTATCAAGG | 60 | 150 |
|  | X5RTR1 | CCAACTTGTTCTGTAGTCTCC | 60 |  |
| *Xa21* | Gene specific pimers |  |  |  |
|  | Xa21.1F | GGGGTGTTTACATCCATAGG/  ATGATATCACTCCCATTATTGC | 60 | 1700 |
|  | Xa21.1R | CGCTCAAGTTGTTTTCGTAGG |  |  |
|  | Xa21.2F | GACCTCGGCGACAACTACCT | 60 | 1000 |
|  | X21.2R | GGCAATCACCAAGCGTGTTAG |  |  |
|  | Xa21EF | GACCTCGGCGACAACTACCT | 61 | 3000 |
|  | Xa21ER | TTCAGCCAGTTCTCAGAATCC |  |  |
|  | Xa21.3F | CAGATACCCACATCCTTAGC | 58 | 1000 |
|  | Xa21.3R | CTCCATCAGTTTCACTAGAGG |  |  |
|  | Xa21.4PF | GCAATGTGCTGTTAGATTCTG | 60 | 1600 |
|  | Xa21.4PR | CACACACGTCAACTAGGAAGC/  TCAGAATTCAAGGCTCCCACC |  |  |
|  | Primer for RT |  |  |  |
|  | X21RTF | GCTCGATGGGATTTATAGGG | 60 | 150 |
|  | Xa21RTR | GCTTCCCGGTTACTATTTCC | 60 |  |
| *Xa26* | Gene specific primers |  |  |  |
|  | Xa26.1F | CAGGGGTTGTGATGTACTGG | 60 | 1400 |
|  | Xa26.1R | GTCGCTGCTGTTACTCTTGC |  |  |
|  | Xa26.2F | GCATACTCTTGCTGCCAATGC | 60 | 1300 |
|  | Xa26.2R | GATCCATCCAGCAAGTTTCC |  |  |
|  | Xa26.3F | AGATTTGACGACGTGCAACC | 58 | 1300 |
|  | Xa26.3R | GTCAGGCTTACCAGCAGAAG |  |  |
|  | Xa26.4F | ATGTGGTGCTGCCCGTTTAGG | 62 | 1300 |
|  | Xa26.4R | CATGTCAAGCTACCAGTGAAGC |  |  |
|  |  |  |  |  |
