# Supplementary figures and images for: Nucleotide Diversity Analysis of Three Major Bacterial Blight Resistance Genes in Rice
Source: PLoS One. 2015 Mar 25;10(3):e0120186. doi: 10.1371/journal.pone.0120186 (PMC4373814; doi:10.1371/journal.pone.0120186)

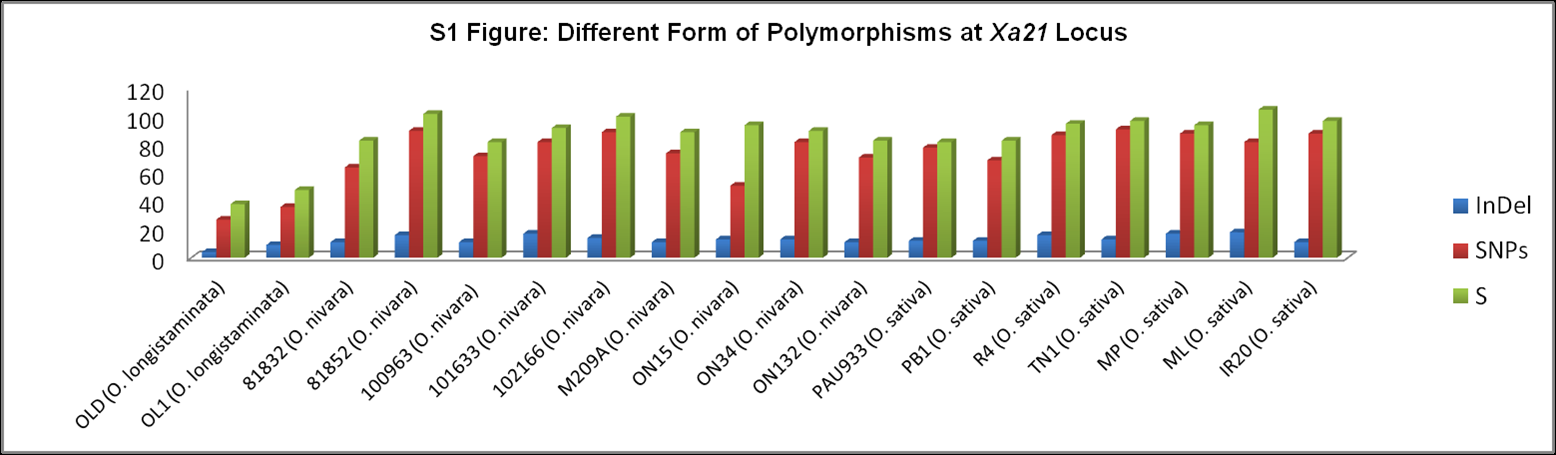

Supplement: S1 Fig — Blue, red and green colors indicate InDels, SNPs and total number of polymorphic sites, respectively. Different accessions are shown in X-axis and number of polymorphisms on Y- axis. (TIF) [file pone.0120186.s002.tif]

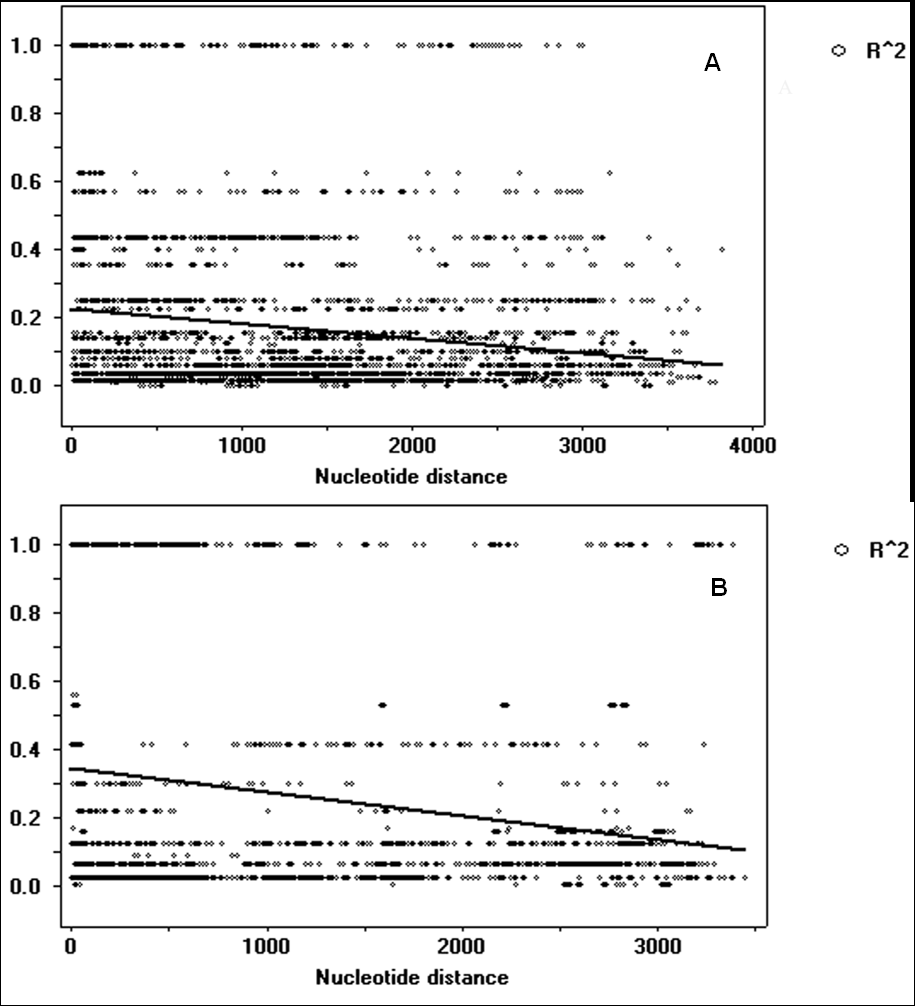

Supplement: S2 Fig — Decay of LD ‘R2’ as a function of distance between pairs of polymorphic sites in Xa21 alleles. The Black line depicts the expected decline of LD against distance based on the equation given by HILL and WEIR (1988). (TIF) [file pone.0120186.s003.tif]

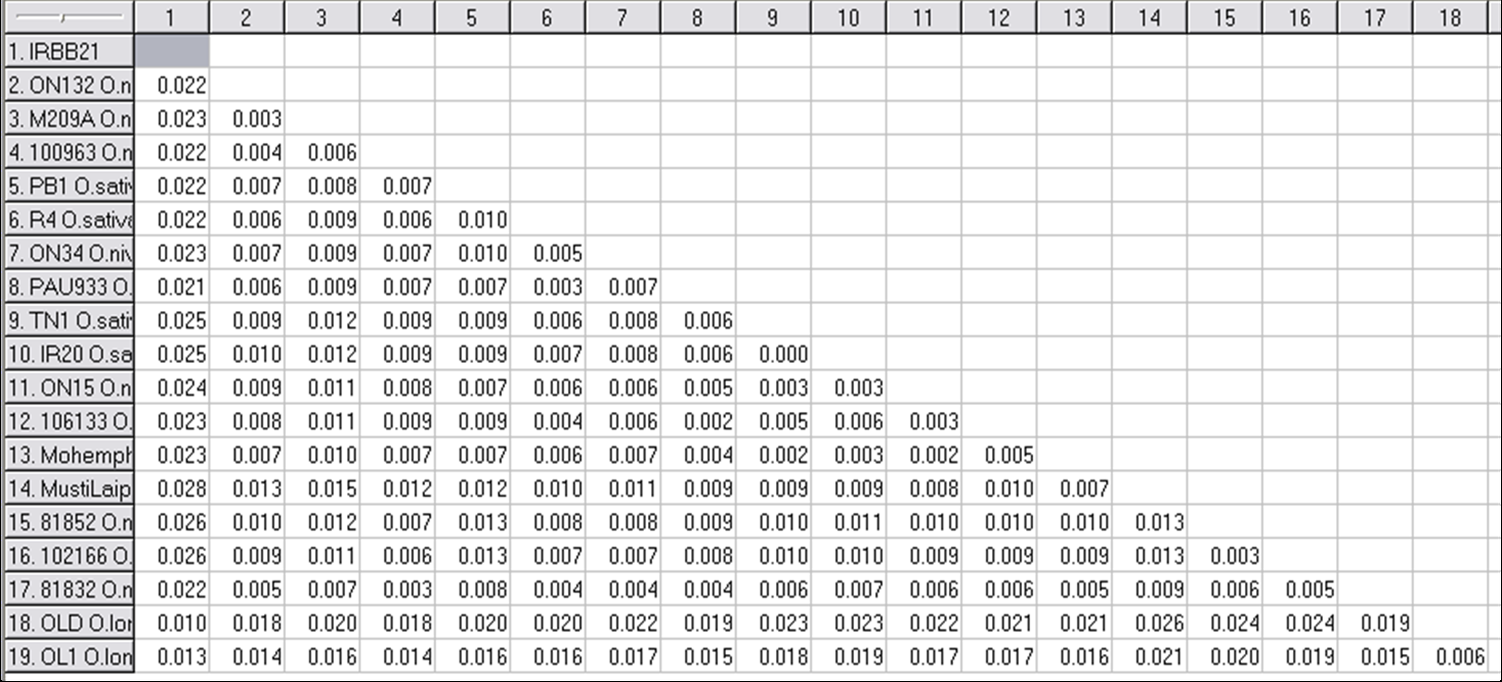

Supplement: S3 Fig — The difference in base composition bias per site is shown in each column. Left column indicate the alleles and the numbering columns indicate the divergence rate corresponding to the numbered rows. (TIFF) [file pone.0120186.s004.tiff]

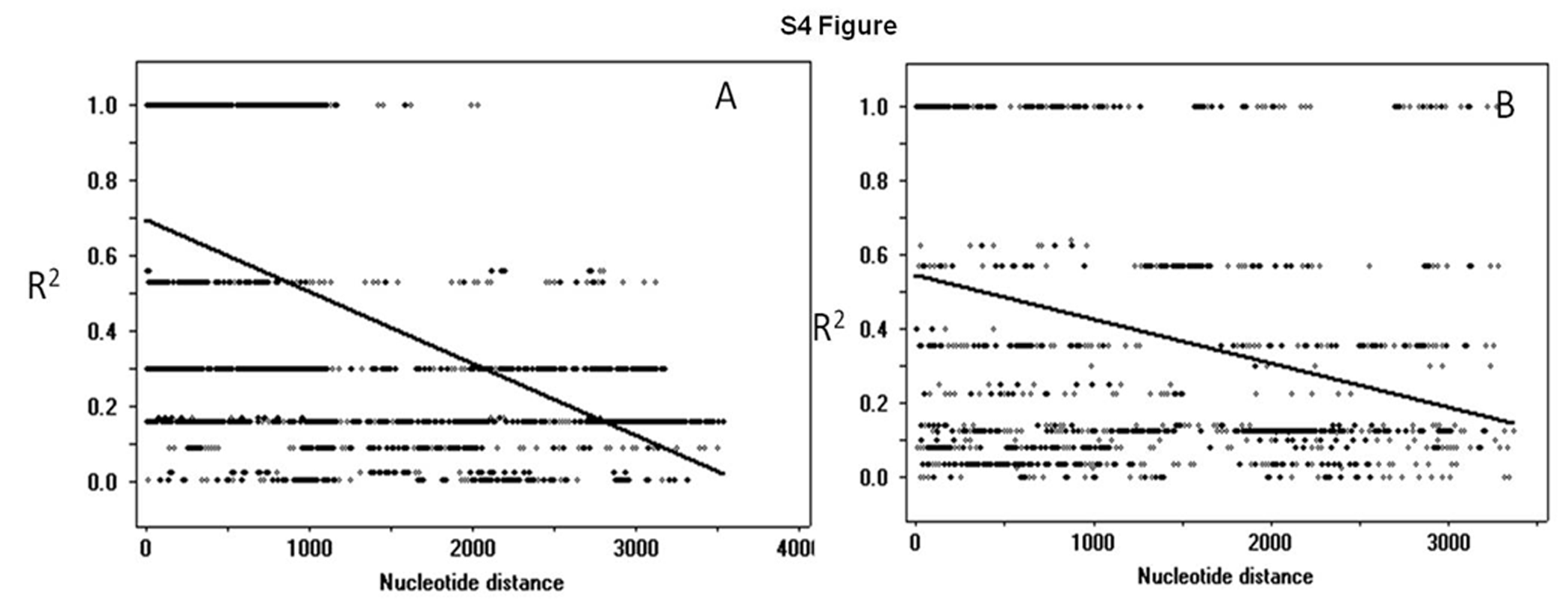

Supplement: S4 Fig — Decay of LD ‘R2’ as a function of distance between pairs of polymorphic sites in Xa26 alleles. The Black line depicts the expected decline of LD against distance based on the equation given by HILL and WEIR (1988). (TIF) [file pone.0120186.s005.tif]

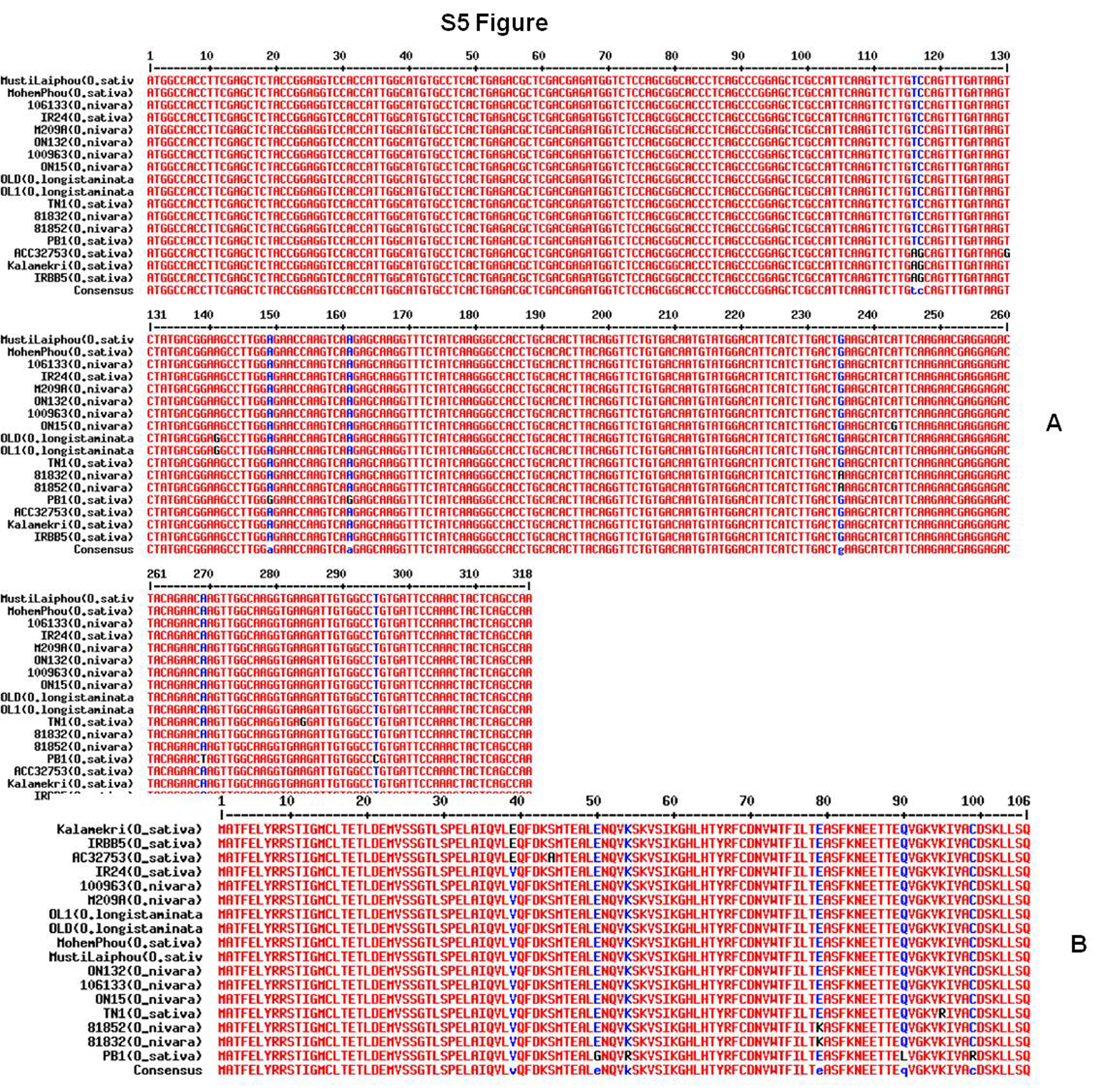

Supplement: S5 Fig — Substitutions in the CDS are represented with different colors. (B) Output of multiple alignment for the predicted amino acid sequences of xa5 alleles. The rice genotypes are indicated in left column. The numbers on the top of the sequences indicate the position of amino acids. Different colors in amino acid shows non synonymous changes. Alignment was performed using Multalin program. (TIF) [file pone.0120186.s006.tif]

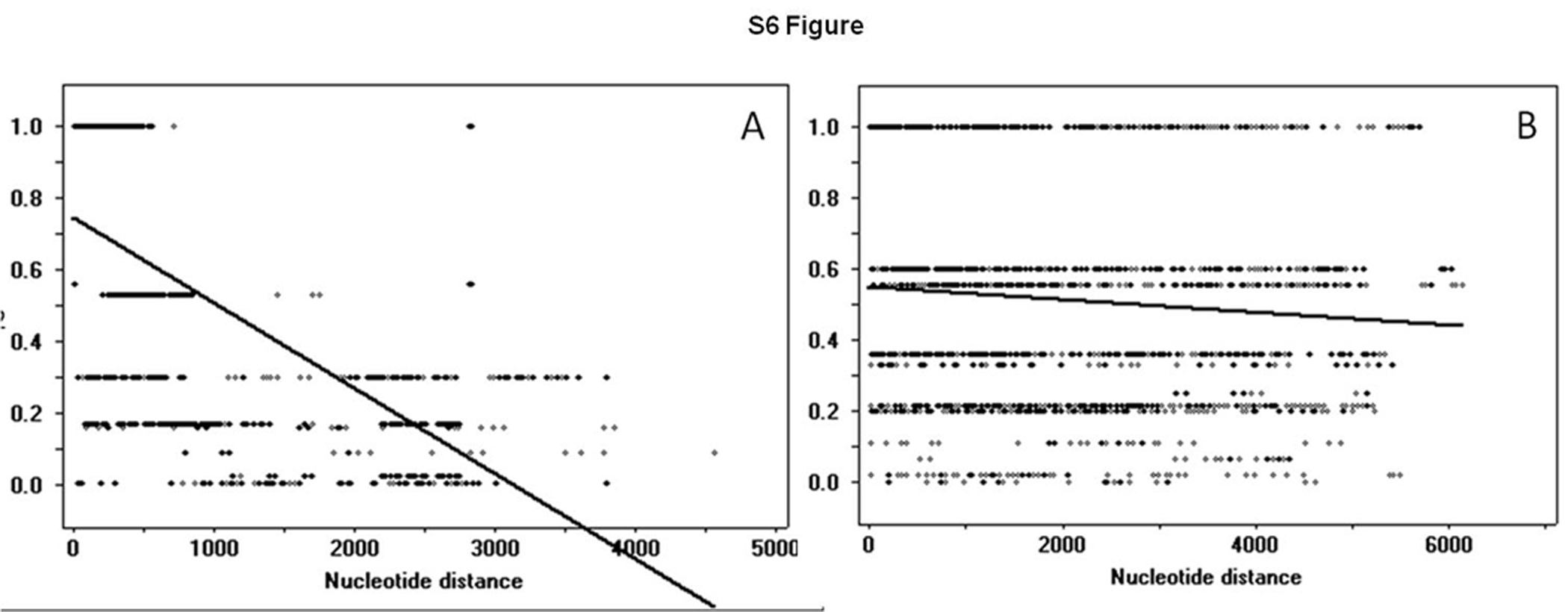

Supplement: S6 Fig — Decay of LD ‘R2’ on X-axis as a function of distance between pairs of polymorphic sites in xa5 alleles and nucleotide distance on Y-axis. The Black line depicts the expected decline of LD against distance based on the equation given by HILL and WEIR (1988). (TIF) [file pone.0120186.s007.tif]

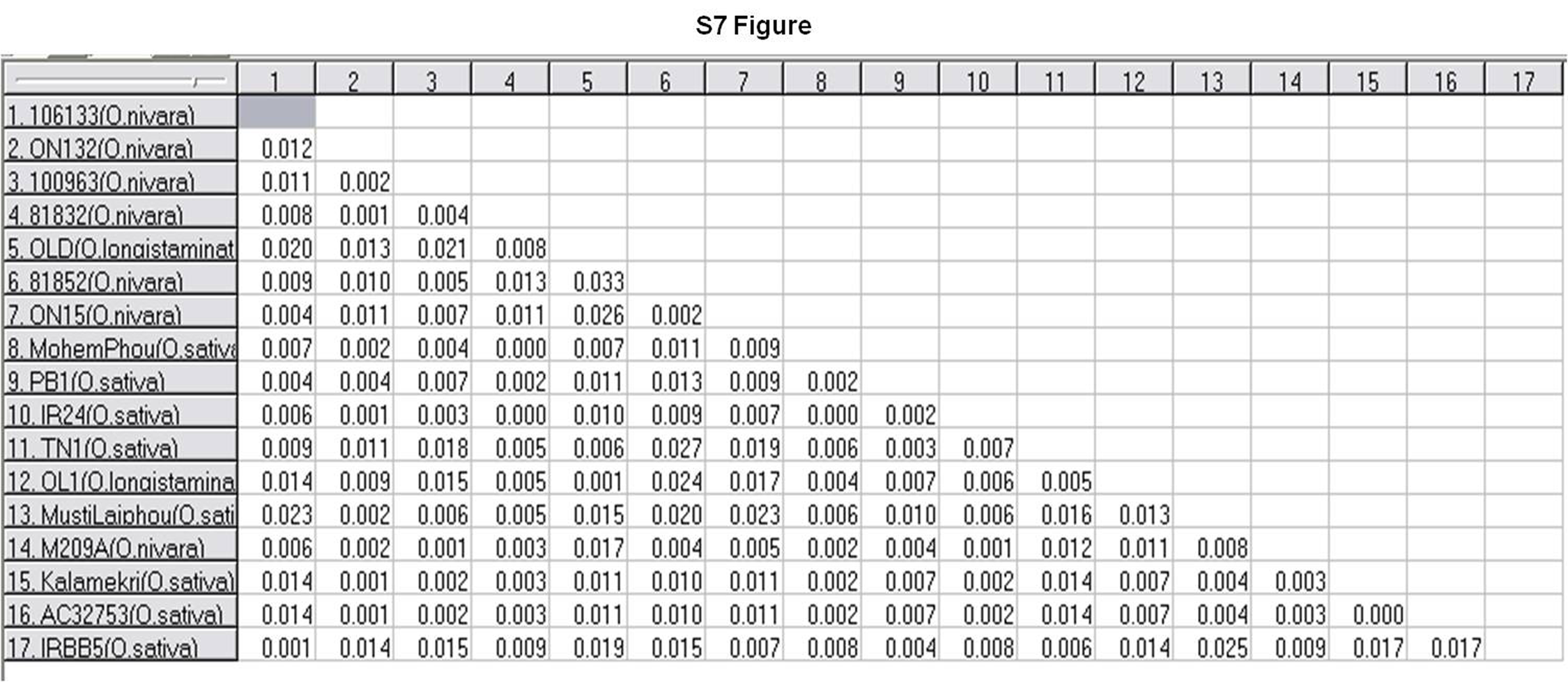

Supplement: S7 Fig — The difference in base composition bias per site is shown in each column. Left column indicate the alleles and the numbering columns indicate the divergence rate corresponding to the numbered rows. (TIF) [file pone.0120186.s008.tif]
